# Optimization of a malachite green–pyrophosphatase assay for the Zika virus RNA-dependent RNA polymerase and preliminary identification of natural product modulators

**DOI:** 10.3389/fddsv.2026.1749327

**Published:** 2026-04-13

**Authors:** Vanessa Aitken, Anthony F. T. Moore, Manuel Terrazas-López, Salvador Rodarte, Eda Koculi

**Affiliations:** 1 Department of Chemistry and Biochemistry, The University of Texas at El Paso, El Paso, TX, United States,; 2 Department of Chemistry, University of Central Florida, Orlando, FL, United States,; 3 Computational Sciences Program, The University of Texas at El Paso, El Paso, TX, United States

**Keywords:** digallic acid, malachite green assay, purpurogallin, RNA-dependent RNA polymerase, Zika virus

## Abstract

The Zika virus (ZIKV) is an *Orthoflavivirus* linked to several neurological and developmental diseases, and it remains a significant global health concern for which no treatments or vaccines are currently available. In this study, we optimized the malachite green colorimetric assay, previously used to measure the RNA-dependent RNA polymerase (RdRp) activity of the hepatitis C and foot-and-mouth disease viruses, and applied this assay for the preliminary identification of the ZIKV RdRp modulators. RdRp is an enzyme essential for viral replication. Modulator screening was conducted using 204 compounds from the Natural Product Set IV of the National Cancer Institute Developmental Therapeutics Program. We identified two compounds, purpurogallin and digallic acid, as preliminary modulators of the ZIKV RdRp. Future studies will validate these preliminary hits using orthogonal assays, perform dose-response analyses, and elucidate their mechanisms of action.

## Introduction

The Zika virus (ZIKV) is a positive-sense, single-stranded RNA virus belonging to the *Orthoflavivirus* genus, which can, in certain cases, cause neurological and developmental disorders (Giraldo et al., 2023; de Sales-Neto et al., 2024; Postler et al., 2023; Godoy et al., 2017). While the primary route of ZIKV transmission is through mosquito bites, it can also be transmitted from mother to fetus and *via* bodily fluids (Giraldo et al., 2023; de Sales-Neto et al., 2024; Godoy et al., 2017; Rabe et al., 2025). Recently, more than 92 countries have experienced a resurgence of ZIKV outbreaks (Rabe et al., 2025). Moreover, rising global temperatures are expected to increase the risk of ZIKV infections, with an estimated 1.3 billion people projected to be at risk by 2050 (Ryan et al., 2019; Ryan et al., 2021). To date, treatment and vaccines for ZIKV are not available (de Sales-Neto et al., 2024). Therefore, the development of new therapeutic agents to treat ZIKV infections is of significant public health interest (de Sales-Neto et al., 2024). In this study, we optimized a previously used malachite green high-throughput screening (HTS) assay and used it to screen for small-molecule modulators of the ZIKV RNA-dependent RNA polymerase (RdRp), using the Natural Product Set IV from the National Cancer Institute’s Developmental Therapeutics Program (Vardakou et al., 2014; Nguyen et al., 2013; Van Veldhoven and Mannaerts, 1987; Tillotson et al., 2017; Moore et al., 2021; Moore et al., 2025; Sivinski et al., 2022). To the best of our knowledge, this assay has not been used previously to search for ZIKV inhibitors.

The ZIKV genome single-stranded RNA functions as an mRNA (Giraldo et al., 2023; de Sales-Neto et al., 2024). From this mRNA all the proteins required for virus structure, replication and propagation are translated (Giraldo et al., 2023; de Sales-Neto et al., 2024). This RNA is translated by the host cellular machinery into a single-polypeptide chain consisting of ten viral proteins: C, preM, E, NS1, NS2A, NS2B, NS3, NS4A, NS4B, and NS5 (Giraldo et al., 2023; de Sales-Neto et al., 2024; Sager et al., 2018). The polypeptide is then cleaved by viral and cellular proteases to produce three structural proteins, C, preM, and E, which form the viral shell. The seven nonstructural proteins, NS1, NS2A, NS2B, NS3, NS4A, NS4B, and NS5 are involved in viral replication and immune evasion (Sager et al., 2018; Sirohi and Kuhn, 2017). NS5 contains two domains connected by a linker: an N-terminal methyltransferase domain and a C-terminal RdRp domain (Flory et al., 2021). RdRp is responsible for replicating the ZIKV RNA genome and is essential for ZIKV replication (Flory et al., 2021). RdRp represents an attractive therapeutic target for treating ZIKV infections with potentially minimal side effects because it is encoded exclusively by the ZIKV genome, humans do not possess any homologous protein with RdRp activity, and it plays a critical role in the ZIKV life cycle (Godoy et al., 2017).

Previously, the malachite green HTS assay was employed to measure RdRp activity from hepatitis C and foot-and-mouth disease viruses (Nguyen et al., 2013). The malachite green is a colorimetric assay that detects the complex formed from malachite green, molybdate, and inorganic phosphate (Vardakou et al., 2014; Van Veldhoven and Mannaerts, 1987; Tillotson et al., 2017; Moore et al., 2021; Moore et al., 2025; Sivinski et al., 2022). However, the RdRp from hepatitis C and foot-and-mouth disease viruses, and polymerases in general, produce pyrophosphate during their reactions, instead of inorganic phosphate (Nguyen et al., 2013). Thus, in the previous study, the pyrophosphatase enzyme, which converts pyrophosphate to inorganic phosphate, was included in the RdRp reaction buffers (Nguyen et al., 2013).

Traditional methods for monitoring polymerase activity include the direct detection of radiolabeled nucleic acid products using gel electrophoresis or thin-layer chromatography (Schrier and Wilson, 1973; Shatkin and Sipe, 1968; Sierra et al., 2007; Rodriguez-Wells et al., 2001; Jin et al., 2012). These approaches are highly sensitive. On the other hand, they require strict regulatory handling and careful disposal due to radiation hazards. More importantly, the radiolabeled methods are not compatible with HTS. Thus, while valuable for hit validation, they are impractical for initial large-scale compound screens.

Non-radioactive alternatives, which involve chromophore-labeled nucleotides incorporated into the polymerized RNA molecule, avoid the safety concerns. However, a shortcoming of these assays is less efficient incorporation of modified nucleotides by RdRp than their natural counterparts (Vassiliou et al., 2000; Park et al., 2002). Thus, an inhibitor may appear to work because the polymerase is inefficient in incorporating the chromophore-labeled nucleotides (Vassiliou et al., 2000; Park et al., 2002).

Luminescence-based assays provide another option: pyrophosphate released during nucleotide incorporation is converted to ATP by ATP sulfurylase in the presence of adenosine 5′-phosphosulfate, and the resulting ATP fuels a luciferase reaction that generates detectable photons (Lahser and Malcolm, 2004). However, practical limitations constrain this assay’s utility in HTS. The luminescence signal typically decays rapidly, requiring precisely timed measurements, and background ATP present in reaction components can contribute to the signal, complicating data interpretation (Lahser and Malcolm, 2004).

In contrast, the coupled malachite green/pyrophosphatase colorimetric assay is simple, inexpensive, non-radioactive, and highly adaptable for HTS (Nguyen et al., 2013). By quantifying inorganic phosphate generated during nucleotide incorporation, it provides a robust and reliable readout of polymerase activity (Nguyen et al., 2013). Its proven reliability across diverse enzyme reactions demonstrates its robustness and reproducibility, making it particularly well suited for HTS of compound libraries (Tillotson et al., 2017; Moore et al., 2021; Moore et al., 2025; Park et al., 2002; Wu, 2011).

In order to examine a large library of compounds and accurately determine the modulation of RdRp activity, it is important to design an HTS assay that is both reproducible from day-to-day and offers an expansive dynamic range. While the malachite green HTS assay previously employed for hepatitis C and foot-and-mouth disease virus RdRp was elegantly designed and executed, key performance metrics, such as dynamic range (Z′) and signal window (SW), were not systematically evaluated (Nguyen et al., 2013; Iversen et al., 2006). In this study, we adapted the malachite green HTS assay and performed statistical analysis using Z′ and SW parameters (Iversen et al., 2006). Next, we used the optimized assay to screen for natural product inhibitors of the Zika virus RdRp, and identified two compounds, purpurogallin and digallic acid, that modulate the ZIKV RdRp activity. In its current form, the malachite green assay described here can be used to identify small-molecule modulators of DNA and RNA polymerases in a high-throughput manner.

## Materials and methods

### Plasmid construction and protein expression

RdRp coding sequence from the ZIKV Puerto Rico strain PRVABC59 was inserted into the pET His6 Sumo TEV LIC (Novagen) by the Protein Production and Analysis Facility at the Sanford Burnham Prebys Medical Discovery Institute in Florida. The final protein construct consisted of a His-tag followed by a SUMO sequence, a TEV protease cut site, eight of the ten amino acid residues of the linker region separating the methyltransferase from RdRp, and the entire sequence of RdRp. The SUMO sequence has been shown to enhance the translation and solubility of proteins expressed in bacteria (Butt et al., 2005). The linker region connecting the methyltransferase and RdRp was retained because this region has been shown to regulate RdRp activity (Flory et al., 2021). The His-tag and SUMO sequence were not cleaved. Thus, the protein assay described here was performed with the RdRp construct containing both the His-tag and SUMO sequence. The inorganic pyrophosphatase from *Saccharomyces cerevisiae* was purchased from Fisher Scientific.

### RdRp expression and purification

*Escherichia coli* (*E. coli*) BL21 CodonPlus (DE3) cells were transformed with the pET-His-SUMO-TEV-LIC vector containing the RdRp coding sequence. The cells were grown in Luria Broth medium supplemented with 30 μg/mL kanamycin and 34 μg/mL chloramphenicol at 37 °C until reaching an optical density at 600 nm (OD_600_) of approximately 0.6. Protein expression was induced with 500 μM isopropyl β-D-1-thiogalactopyranoside, and the cultures were incubated for an additional 16 h at 20 °C. Cells were harvested by centrifugation.

The cell pellets were resuspended in a lysis buffer consisting of a phosphate-buffered saline (PBS: 137 mM NaCl, 2.7 mM KCl, 10 mM Na_2_HPO_4_, 1.8 mM KH_2_PO_4_), supplemented with 5 mM β-mercaptoethanol (BME) and 1 mM phenylmethylsulfonyl fluoride (PMSF). Cells were lysed by sonication on ice using a Branson Digital Sonifier 450 equipped with a 0.5 mm diameter tip using six 30-s pulses in ice. The lysate was cleared by centrifugation at 11,648 × g for 60 min at 4 °C.

The clarified supernatant was loaded onto an 8 mL Ni-NTA-affinity column pre-equilibrated with PBS containing 5 mM BME, 1 mM PMSF and 20 mM imidazole. The protein was eluted using PBS supplemented with 5 mM BME, 1 mM PMSF and 500 mM imidazole. Fractions containing the RdRp protein were pooled and diluted 1:10 with a buffer consisting of 25 mM Tris-HCl (pH 8.0) and 5 mM BME.

Subsequently, the protein sample was loaded onto a 5 mL heparin affinity column (HiTrap Heparin HP, GE Healthcare Life Sciences). Heparin columns selectively bind nucleic acid–binding proteins and compete with nucleic acids for protein interaction sites (Gao et al., 2021). Therefore, this step facilitated both the further purification of the RdRp protein and the removal of any bound nucleic acids. The column was equilibrated and washed with a buffer consisting of 25 mM Tris-HCl (pH 8.0), 30 mM NaCl, and 5 mM BME. The RdRp was eluted using a linear gradient of the heparin elution buffer consisting of 25 mM Tris-HCl (pH 8.0), 1000 mM NaCl, and 5 mM BME.

To further remove undesired proteins and RdRp aggregates, fractions containing the RdRp were pooled, concentrated, and loaded onto a size-exclusion chromatography column (Superdex 200 10/300 GL, GE Healthcare). The column was equilibrated with the gel-filtration buffer consisting of 25 mM Tris-HCl (pH 8.0), 100 mM KCl, and 2 mM dithiothreitol (DTT). Fractions containing the RdRp were combined and concentrated using 10 kDa molecular weight cutoff centrifugal filters (Amicon Ultra Ultracel-10K, Millipore). Finally, purified RdRp was flash-frozen in small aliquots and stored at −80 °C until use.

### Using the malachite green assay for screening inhibitors of the ZIKV RdRp

The RNA polymerase reaction conditions were adapted from a previous study (Vardakou et al., 2014). Pyrophosphate release during the RdRp reaction was measured using the malachite green/molybdate colorimetric assay (Vardakou et al., 2014). Because the malachite green/molybdate colorimetric assay specifically detects inorganic phosphate, but not pyrophosphate, an inorganic pyrophosphatase was added to the reaction mixture to convert a pyrophosphate into an inorganic phosphate (Vardakou et al., 2014; Nguyen et al., 2013; Van Veldhoven and Mannaerts, 1987).

The RNA polymerase reaction initiation complex was formed by annealing poly(C) RNA molecules (200–250 nucleotides in length) with a 10-nucleotide-long poly(G) RNA primer (Lu et al., 2017). The poly(C) RNA was purchased from Sigma-Aldrich and further purified by polyacrylamide gel electrophoresis (Moore et al., 2025; Moore et al., 2017; Woodson and Koculi, 2009; Childs et al., 2016). The HPLC-purified poly(G) oligonucleotides were obtained from Integrated DNA Technologies (IDT). Annealing was performed by mixing the two RNA strands in an annealing buffer containing 10 mM Tris-HCl (pH 7.5), 100 mM NaCl, and 0.2 mM EDTA-KOH (pH 8.0). The mixture was incubated at 95 °C for 2 min and then cooled slowly to room temperature, in order to promote proper duplex formation (Moore et al., 2025).

The RdRp reaction was performed in a 384-well plate at 22 °C, in a 12.5 μL reaction mixture consisting of 5 mM Tris-HCl pH 7.5, 7 mM NaCl, 8 mM MnCl_2_, 10% glycerol, 0.01% Triton X-100, 10 mM DTT, 0.05 mU/μL inorganic pyrophosphatase, 0.5 mU/μL SUPERase·In, 500 nM annealed RNA, 0.5 mM GTP·Mn, 0–200 nM RdRp, and 1% DMSO (Lu et al., 2017). Reactions for compound screening also contained 10 μM of compound. Because the objective of this study was to optimize, validate, and assess the feasibility of the malachite green–pyrophosphatase assay, only a single polymerase reaction was performed for each small molecule. Replicate confirmation was beyond the scope of this study. The RdRp reactions were initiated by the addition of GTP Mn and allowed to continue for 30–90 min. These reactions were quenched with 10 mM EDTA, which sequesters Mn^2+^ and halts the RdRp reaction (Moore et al., 2025; Lu et al., 2017).

To detect the orthophosphate generated by pyrophosphatase activity, 75 μL of the malachite green–molybdate reagent was added to the RdRp reaction mixture. After 1 min, 10 μL of 34% sodium citrate was added to stop the reaction and stabilize the color complex. The mixture was then incubated at 22 °C for 50 min before measuring absorbance at 625 nm using a Molecular Devices SpectraMax Plus 384 spectrophotometer and microplate reader. The malachite green–molybdate reagent was prepared as previously described (Moore et al., 2021; Moore et al., 2025).

(1) $${\bar{X}}_{p}-3\sigma_{p}$$

Compounds were classified as modulators if their normalized activity fell below. In Equation 1, ${\bar{\text{X}}}_{\text{p}}$ is the average absorbance of the positive control, while σ_p_ is the standard deviation of the ${\bar{\text{X}}}_{\text{p}}$. Equation 1 ensures that selected hits differed from the positive control by at least three standard deviations, corresponding to a 99.7% confidence level under a normal distribution (Pukelsheim, 1994).

For the two compounds classified as modulators, the RdRp reaction absorbance value in their presence was normalized to the RdRp positive and negative controls reactions averages. For this normalization, the ${\bar{\text{X}}}_{\text{p}}$ was defined as 100% enzymatic activity, and the average absorbance of the negative control $\left({\bar{\text{X}}}_{\text{n}}\right)$ reactions defined as 0% activity. Normalized activity (%) for the two compounds was calculated using the equation below:

(2) $$\text{Normalized}\;\text{Activity}\;(\%)=\frac{\left(C_{\text{compound}}-{\bar{X}}_{n}\right)}{\left({\bar{X}}_{p}-{\bar{X}}_{n}\right)}\times 100$$

where *C*_*compound*_ is the absorbance value of the RdRp reaction in the presence of the compound.

## Results and discussion

### Optimization of the malachite green assay for ZIKV RdRp inhibitor screening

**FIGURE 1:**
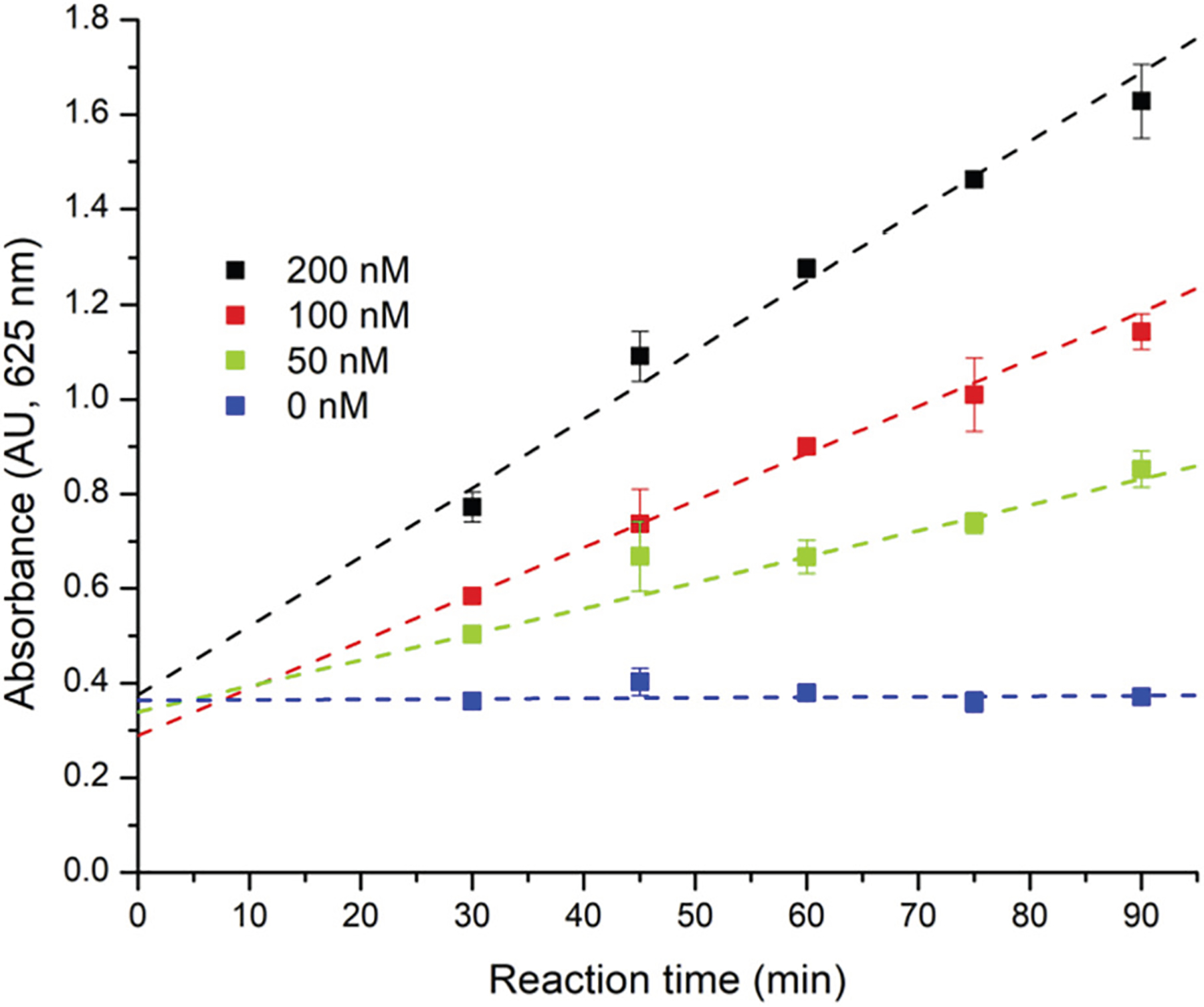
The malachite green assay used to measure RdRp activity is linear across a range of enzyme concentrations and reaction times. Dotted lines represent linear fits of the data for each RdRp concentration over increasing reaction times. Assays performed in the absence of RdRp are shown as blue squares; with 50 nM RdRp as green squares; 100 nM RdRp as red squares; and 200 nM RdRp as black squares. Data points represent the mean of three independent experiments. Error bars indicate standard deviations. Based on these results, a 75-min reaction with 200 nM RdRp was selected for inhibitor screening to maximize signal strengths while maintaining assay linearity.

The malachite green assay quantifies orthophosphate by detecting the green-colored complex formed from orthophosphate, malachite green, and molybdate (Van Veldhoven and Mannaerts, 1987; Tillotson et al., 2017; Moore et al., 2021; Moore et al., 2025; Sivinski et al., 2022). However, during double-stranded RNA synthesis, the RdRp releases a pyrophosphate as a byproduct (Nguyen et al., 2013). To enable the detection of the pyrophosphate using the assay, an inorganic pyrophosphatase, which converts a pyrophosphate to an orthophosphate, was included in the reaction mixture (Vardakou et al., 2014; Nguyen et al., 2013). We aimed to determine the RdRp reaction and assay conditions that yield reliable, proportional, and accurate measurements. Therefore, we evaluated the linearity of the RdRp reaction, as measured by the malachite green assay, with respect to both time and RdRp concentration. Our results demonstrate that the assay response remains linear for at least 90 min (Figure 1). Based on these findings, we selected a 75-min reaction time with 200 nM RdRp to maximize signal intensity while maintaining assay linearity.

### Robustness, reproducibility, and dynamic range of the malachite green assay employed in this study

**FIGURE 2:**
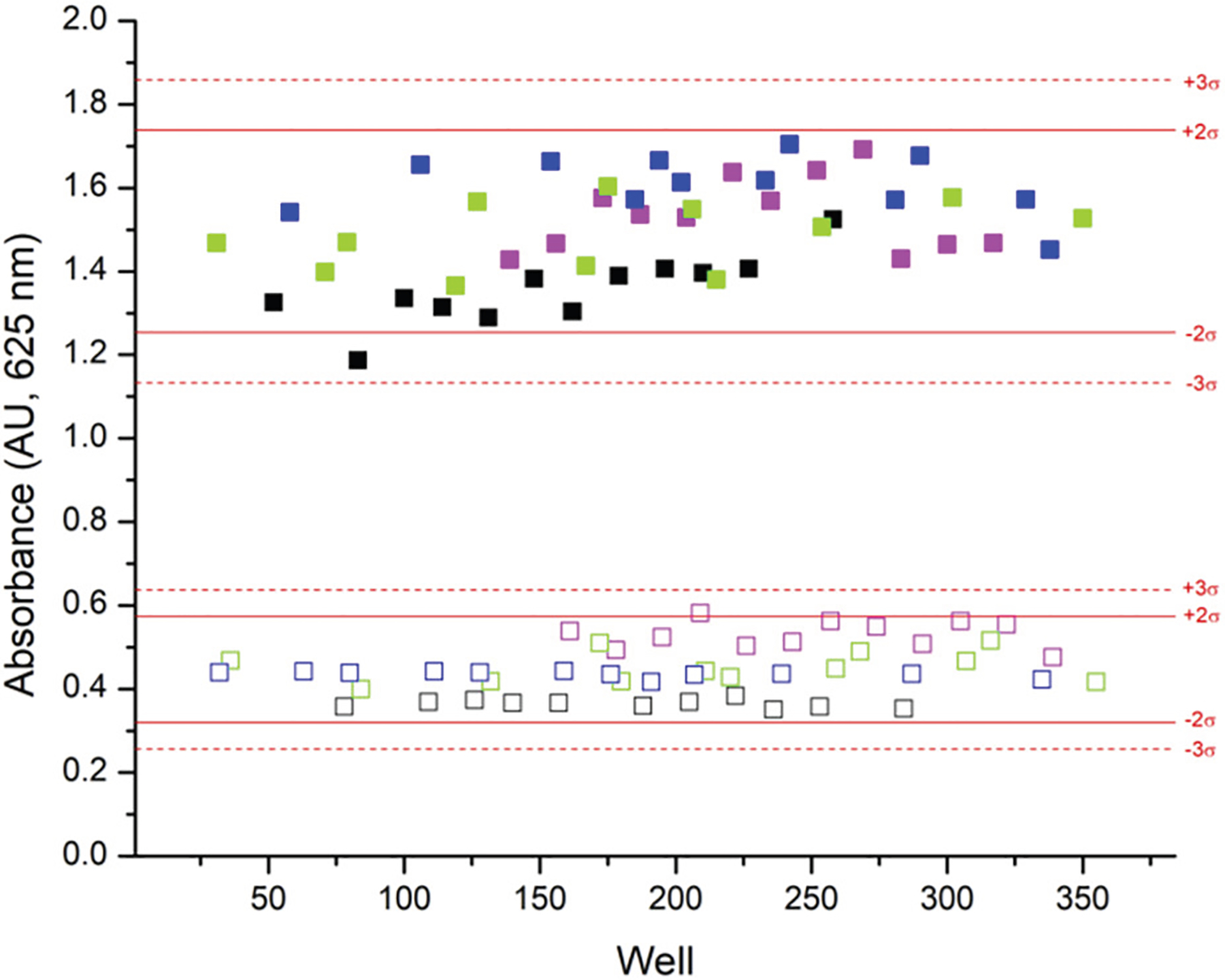
The malachite green HTS assay is reproducible across multiple days. Absorbance measurements were obtained for positive and negative controls using 384-well microplates across four independent assay days. Each color represents a different day (Days 1, 2, 3, and 4). Positive controls, containing the ZIKV RdRp and RNA substrate, are represented by filled squares, and negative controls, lacking the enzyme, by empty squares. σ denotes the standard deviation from the mean absorbance values. The solid and dotted red lines indicate two and three standard deviations, respectively, from the mean of the positive or negative control groups.

HTS robustness testing ensures that minor variations in experimental conditions across different days do not compromise the accuracy of hit detection. The hit detection can be compromised by misidentifying false positives as true hits, or by failing to detect true positives. These evaluations are essential for maintaining consistency and reliability in assay performance and ensuring that inter-day variability remains minimal. To assess the robustness of the malachite green HTS assay used for evaluating ZIKV RdRp activity, absorbance signals were measured for both positive and negative controls of the RdRp reactions. Negative controls lacked the RdRp enzyme, while positive controls contained both the RNA substrate and the enzyme. To evaluate reproducibility, the assays were conducted on four separate days. Our results showed minimal variation in absorbance values across both positive and negative RdRp control reactions. Most of the measurements fell within two standard deviations of ${\bar{\text{X}}}_{\text{p}}$ and ${\bar{\text{X}}}_{\text{n}}$, and all of the values fell within three standard deviations of the mean absorbance value (Figure 2).

The Z′ factor is a dimensionless statistical parameter used to assess the signal dynamic range and variability between the positive and negative controls in an HTS assay (Equation 3) (Iversen et al., 2006). A high Z′ value indicates strong separation between positive and negative control signals and low data noise. Both strong separation between positive and negative controls and low data noise are essential for distinguishing true hits from experimental variability. An acceptable Z′ value ranges from 0.5 to 1 (Iversen et al., 2006). All Z′ values determined from experiments conducted over four separate days fell within the above range, indicating that malachite green assay as optimized by us is robust, exhibits a high signal-to-noise ratio, and is suitable for HTS (Table 1).


(3) $$Z\prime =1-\frac{\left(3\sigma_{p}+3\sigma_{n}\right)}{{\bar{X}}_{p}-{\bar{X}}_{n}}$$

In Equation 3, ${\bar{\text{X}}}_{\text{p}}$, ${\bar{\text{X}}}_{\text{n}}$, and σ_p_, are the parameters defined in Equations 1, 2, while σ_n_ is the standard deviation of ${\bar{\text{X}}}_{\text{n}}$ (Iversen et al., 2006).

SW is a dimensionless parameter used to assess the difference in signal between negative and positive controls (Iversen et al., 2006). SW was calculated using Equation 4 across four different days and plates (Table 1) (Iversen et al., 2006). An SW value greater than two is considered adequate for detecting inhibitors in an HTS assay. The SW values obtained on different days were significantly greater than 2, demonstrating once again that our malachite green HTS assay is both robust and sensitive (Iversen et al., 2006).


(4) $$\text{SW}=\frac{{\bar{X}}_{p}-{\bar{X}}_{n}-3(\sigma_{p}+\sigma_{n})}{\sigma_{p}}$$

### Purpurogallin and digallic acid inhibit RdRp activity

The optimized green/molybdate colorimetric assay was used to screen for RdRp inhibitors. A total of 204 out of 419 natural products from the NCI Natural Products Set IV were screened. The screening was performed in the presence of 10 μM of each compound (Supplementary Table S1). The present study is focused on assay optimization, validation and demonstration of feasibility (Figures 1, 2; Table 1). Therefore, only a representative feasibility screening of 204 compounds was performed. An expanded library will be assessed in future work.

**FIGURE 3:**
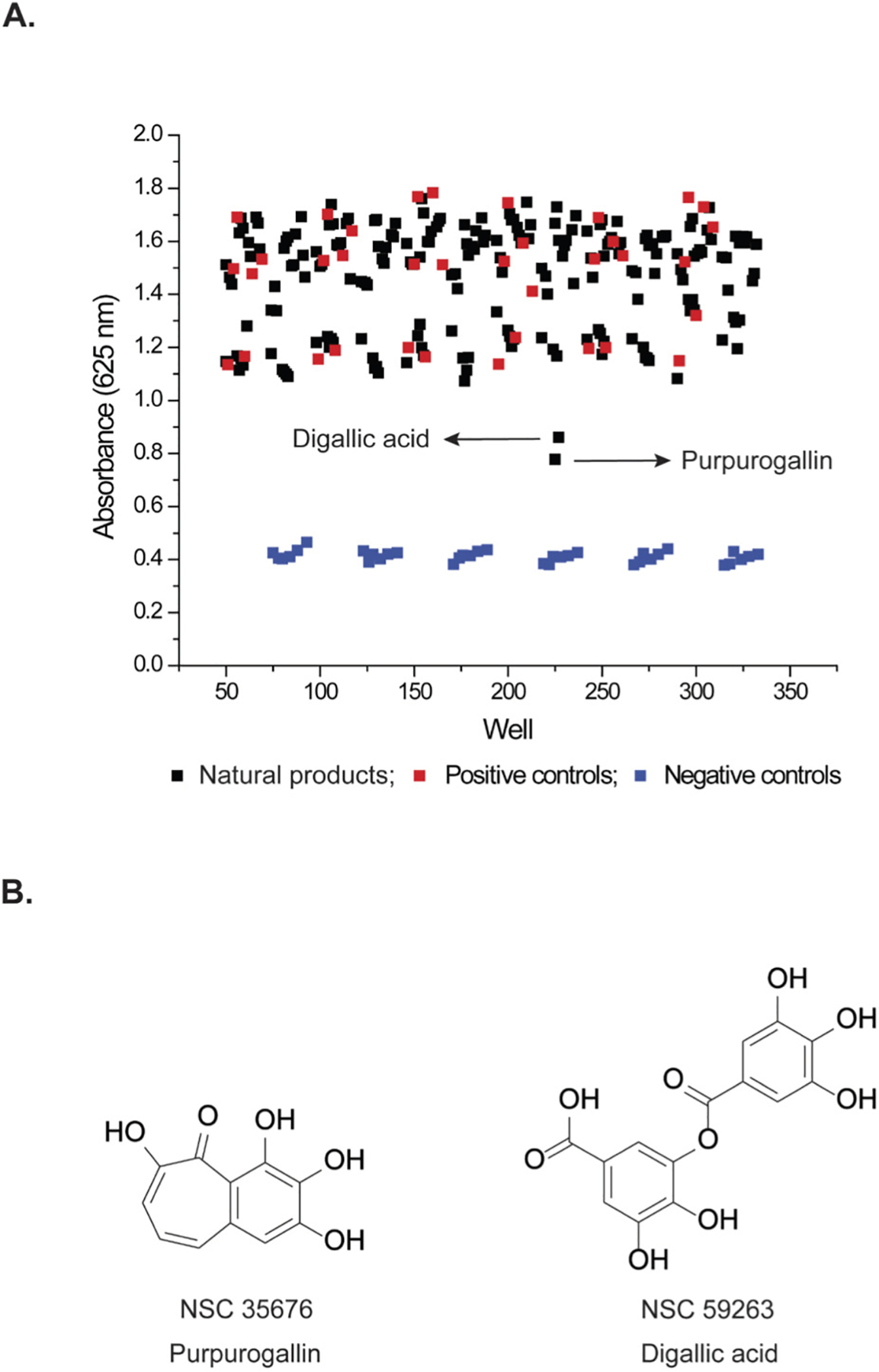
Purpurogallin and digallic acid modulate the polymerase activity of ZIKV RdRp as determined by the malachite green HTS assay. **(A)** A total of 204 out of 419 compounds from the NCI Natural Products Set IV were screened using the malachite green assay. The list of natural products tested is provided in Supplementary Table S1. The absorbance of the positive control reactions, which contained the RNA substrate and the RdRp, is shown as red squares; that of the negative control reactions, which lacked the RdRp enzyme, as blue squares; and the absorbance of the reactions that contained one of the 204 compounds, along with both RNA and RdRp, as black squares. Purpurogallin (NSC 35676) and digallic acid (NSC 59263), which, as determined by our threshold, inhibited the polymerase activity of RdRp are labeled (Equation 1; Table 2). The rest of the investigated compounds, which do not inhibit the activity of RdRp based on our threshold, are not marked. **(B)** Chemical structures of the two compounds that inhibit the polymerase activity of RdRp: purpurogallin (NSC 35676) and digallic acid (NSC 59263).

We found that purpurogallin (National Service Center (NSC) 35,676) and digallic acid (NSC 59263) modulated the polymerase activity of the ZIKV RdRp based on our threshold of 3σ cutoff (Figure 3; Equation 1; Table 2). While any compound can potentially interfere with a colorimetric assay, such as the malachite green, phytochemicals such as digallic acid and purpurogallin, due to their structural complexity and functional groups, could be more prone to affecting colorimetric assays (Figure 3B). Therefore, these digallic acid and purpurogallin are presented as preliminary RdRp modulator candidates, and the potential limitations of our screening have been discussed in detail further in the manuscript.

Purpurogallin is a benzotropolone derivative (Bentley, 2008). This compound occurs naturally in the nut galls of the *Quercus* species or can be synthesized *via* pyrogallol oxidation (Bentley, 2008). Purpurogallin has been shown to protect cells from hydrogen peroxide-induced DNA damage and apoptosis, both of which are associated with oxidative stress (Bentley, 2008). Importantly, ZIKV infection induces oxidative stress, which facilitates viral replication (Kayesh et al., 2025). Therefore, purpurogallin may serve as a dual-function inhibitor by targeting ZIKV RdRp and by mitigating oxidative stress associated with infection.

Moreover, purpurogallin has demonstrated anti-inflammatory effects in human endothelial cells (Kim et al., 2012; Park et al., 2013; Ku et al., 2013). Acute inflammation is considered a central mechanism underlying ZIKV-associated pathology. Hence, modulation of the cellular inflammatory response has been proposed as a promising therapeutic strategy for ZIKV infection (de Sales-Neto et al., 2024). Importantly, the anti-inflammatory compound MK-591 has emerged as a potential candidate for the treatment of ZIKV infection (Paul et al., 2024). Accordingly, purpurogallin may function as a multifunctional inhibitor by attenuating the inflammatory response, inhibiting RdRp activity, and reducing oxidative stress.

The other compound shown to modulate the activity of ZIKV RdRp is digallic acid (Figure 3; Table 2). Digallic acid is derived from *Pistacia lentiscus* fruits or synthesized in laboratory *via* partial hydrolysis of tannic acid (Bhouri et al., 2010; Verzele et al., 1983). A previous study also reported that digallic acid inhibits the reverse transcriptase activity of the murine leukemia virus (MLV) and the human immunodeficiency virus (HIV) (Nakane et al., 1990). In that study, digallic acid inhibited the activity of reverse transcriptase by competing for the template RNA binding site (Nakane et al., 1990). In addition to inhibiting MLV and HIV reverse transcriptase, digallic acid was shown to moderately inhibit the activity of human DNA polymerases alpha and beta, but did not inhibit human DNA polymerase gamma or *E. coli* DNA polymerase I (Nakane et al., 1990). These cross-polymerase inhibitions, combined with mechanistic studies, suggest that digallic acid may interact with conserved structural motifs involved in DNA or RNA template binding. Future structural studies of the ZIKV RdRp and HIV reverse transcriptase in the presence of digallic acid could aid in the design of inhibitors structurally similar to digallic acid. These inhibitors could be highly specific for ZIKV and HIV polymerases and spare the human DNA polymerases. These dual-acting inhibitors may hold therapeutic potential for treating coinfections caused by ZIKV and HIV.

To further evaluate the mechanism of inhibition, we considered whether antioxidant properties might contribute to the observed decrease on the RdRp activity in the presence of purpurogallin and digallic acid. Thus, we compared the inhibitory activity of digallic acid and purpurogallin to that of other natural products tested that possessed antioxidant activity (Supplementary Table S2). Of the 204 compounds investigated, 14 were identified as having antioxidant properties (Supplementary Table S1). With the exception of digallic acid and purpurogallin, none of these antioxidants modulated ZIKV RdRp activity (Supplementary Table S1; Figure 3; Table 2). Therefore, the observed modulation by digallic acid and purpurogallin is unlikely to result from their general antioxidant properties and instead indicates a more specific interaction with the RdRp enzyme.

Despite their promising inhibitory activity, both digallic acid and purpurogallin present limitations in terms of stability and selectivity. Purpurogallin is generally stable under physiological conditions but may undergo degradation when exposed to elevated oxidative or radiolytic stress (Prasad et al., 1994; Ratti et al., 2023). In contrast, digallic acid is not very stable in cellular environments and is susceptible to hydrolysis into gallic acid monomers (Zheng et al., 2024; Rodriguez-Duran et al., 2011; Erdogan and Karadag, 2006). Furthermore, because of their potential for non-specific interactions and promiscuous binding, both compounds present limitations in their specificity for ZIKV RdRp (Bhouri et al., 2010; Ku and Bae, 2014; Veser, 1987; Souto et al., 2020; Zhen et al., 2019; Richard, 2019; Glanzer et al., 2016; Alfonso et al., 2022; Chatterjee et al., 2015; Namkung et al., 2011; Suzuki et al., 2014; Kumar et al., 2024). Nevertheless, these molecules may serve as preliminary chemical scaffolds for further inhibitor developments. Future computational modeling and structural studies of RdRp in complex with purpurogallin or digallic acid may inform rational scaffold optimization, which could improve both stability of these compounds and their selectivity for ZIKV RdRp. The findings presented here provide an early foundation for the development of two new classes of inhibitors.

## Conclusions

The optimized malachite green–pyrophosphatase assay we employed here is robust, reproducible, and sensitive across different experimental days (Table 1). This assay, as optimized by our team, could be employed for the HTS of DNA and RNA polymerases, as well as other enzymes that produce pyrophosphate during their catalytic cycles. Through the screening of NCI natural product libraries, purpurogallin and digallic acid were found as preliminary inhibitors of the activity of the RdRp (Figure 3; Supplementary Table S1).

## Limitations

Because the primary objectives of this work were to optimize, validate, and assess the feasibility of the malachite green–pyrophosphatase assay for HTS rather than to identify or characterize lead inhibitors for clinical development, orthogonal assays were not included, and all compounds were tested only once at a single screening concentration (10 μM). Thus, assay interference cannot be excluded.

In addition, because the objectives of this study were the optimization, validation, and feasibility assessment of the malachite green–pyrophosphatase assay for screening ZIKV RdRp inhibitors, the preliminary inhibitory activity of purpurogallin and digallic acid was evaluated only under *in vitro* conditions. Lastly, the determinations of the purpurogallin and digallic acid concentrations required to inhibit 50% of ZIKV RdRp activity (IC_50_), the measurements of these chemicals cellular antiviral efficacy, and cytotoxicity, remain to be performed.

Future studies will validate the preliminary hits, purpurogallin and digallic acid (Figure 3; Table 2), using assays that directly monitor RdRp-mediated RNA synthesis. In addition, these studies will characterize the IC_50_ values, evaluate antiviral activity in ZIKV-infected cell cultures, and determine the cytotoxicity of purpurogallin and digallic acid in various human cell lines.

## Supplementary Material

SI

The Supplementary Material for this article can be found online at: https://www.frontiersin.org/articles/10.3389/fddsv.2026.1749327/full#supplementary-material

## Figures and Tables

**TABLE 1:** The malachite green HTS assay is robust, has a large dynamic range, and a high signal-to-noise ratio.

Plate/Day^a^	Z′^b^	SW^c^
1	0.731	9.115
2	0.655	7.796
3	0.666	8.790
4	0.807	13.911

a The malachite-green assay, with the negative and positive controls, was performed on different plates on four different days. The positive control reactions contained both the RNA, substrate and the RdRp, while the negative control reactions lacked the RdRp enzyme.

b Z’ values were calculated from experiments performed on four different days using Equation 3. The Z′ values indicate that the separation between the negative and positive controls is sufficient to reliably detect inhibitors. The Z′ values obtained across all 4 days of experimentation fall within the acceptable range of 0.5 < Z′ < 1, demonstrating that the assay exhibits high quality and is suitable for HTS (Iversen et al., 2006).

c SW was calculated using Equation 4 for four different plates across multiple days. The SW values are significantly larger than 2 (the accepted SW, value for an HTS-suitable assay) (Iversen et al., 2006).

**TABLE 2:** Summary of compounds exhibiting significant inhibition of ZIKV RdRp in the malachite green assay.

Compound^a^	Raw signal^b^	${\bar{\text{X}}}_{\text{p}}$ ^a^	${\bar{\text{X}}}_{\text{n}}$ ^c^	Normalized Activity %^d^	$\sigma_{\text{p}}$ ^a^	$3\sigma_{\text{p}}$ cutoff^a^
Digallic acid	0.861	1.213	0.406	56%	0.054	1.015
Purpurogallin	0.777	1.213	0.406	46%	0.054	1.015

a Modulators were defined as compounds with raw signals below ${\bar{\text{X}}}_{p}-3\sigma_{p}$, as described in Equation 1. ${\bar{\text{X}}}_{p}$ is the average signal of the positive controls, and *σ_p_* is the standard deviation of ${\bar{\text{X}}}_{p}$.

b Raw signal corresponds to the RdRp reaction malachite green absorbance measured in the presence of the compound.

c ${\bar{X}}_{n}$ represents the average absorbance signal of the negative controls.

d Normalized activity was calculated as described in Equation 2.

## Data Availability

The original contributions presented in the study are included in the article/Supplementary Material, further inquiries can be directed to the corresponding author/s.
