# Supplementary material for: Optimization of a malachite green–pyrophosphatase assay for the Zika virus RNA-dependent RNA polymerase and preliminary identification of natural product modulators
Source: Front Drug Discov (Lausanne). Author manuscript; Available in PMC 2026 Aug 11. (PMC13455045; doi:10.3389/fddsv.2026.1749327)
Supplement: Supplementary file 1 [file NIHMS2199595-supplement-SI.pdf]

Optimization of a Malachite Green–Pyrophosphatase Assay for the Zika Virus RNA-Dependent RNA Polymerase and Preliminary Identification of Natural Product Modulators

Vanessa Aitken[1], Anthony F. T. Moore[2a], Manuel Terrazas-López[1], Salvador Rodarte[3] and Eda Koculi[1]*

[1]Department of Chemistry and Biochemistry, The University of Texas at El Paso, El Paso, TX 79968, USA

[2]Department of Chemistry, University of Central Florida, 4111 Libra Drive, Physical Sciences, Orlando, FL 32816, USA

[3]Computational Sciences Program, The University of Texas at El Paso, El Paso, TX 79968, USA

*To whom the correspondence should be addressed.

Email: ekoculi@utep.edu

[a] Anthony F.T. Moore has passed away.

Table S1. Comprehensive list of National Service Center (NSC) compounds investigated in this study with structural classifications and COCONUT identifiers[1].

NSC Number[a]	Compound Name[b]	Absorbance (625 nm)[c]	Pathway[d]	SuperClass[d]	Class[d]	COCONUT id[e1]
757	Colchicine	1.15	Alkaloids	Tyrosine alkaloids	Phenethylisoquinoline alkaloids	CNP0215656.1
824	Camphoric acid, (-)-	1.66	Terpenoids	Monoterpenoids	Carane monoterpenoids	CNP0193313.1
1115	Quinate	1.60	Shikimates and Phenylpropanoids	Phenolic acids (C6-C1)	Shikimic acids and derivatives	CNP0205647.2
2080	(+)-Melezitose	1.61	Carbohydrates	Saccharides	Disaccharides	CNP0198142.1
2150	o-Vanillin	1.46	Shikimates and Phenylpropanoids	Phenylpropanoids (C6-C3)	Cinnamic acids and derivatives	CNP0274414.0
2347	1,2,3-Propanetricarboxylic acid	1.67	Fatty acids	Fatty Acids and Conjugates	Dicarboxylic acids	CNP0346076.0
2802	Pinic acid	1.62	Terpenoids	Monoterpenoids	-	CNP0244968.4
2835	Ricinoleic Acid, (+)-	1.70	Fatty acids	Fatty Acids and Conjugates	Hydroxy fatty acids	CNP0311322.3
2952	Dehydroabietic Acid	1.22	Terpenoids	Diterpenoids	Abietane diterpenoids	CNP0192451.2
3053	Dactinomycin	1.14	Amino acids and Peptides	Oligopeptides	Actinomycins	CNP0226661.2
3071	2-Methoxy-5-methyl-1,4-benzoquinone	1.62	Polyketides	Aromatic polyketides	Benzoquinones	CNP0269462.0
3590	Leucovorin Calcium	1.59	Alkaloids	-	-	CNP0567692.5
3716	Levulinic Acid	1.51	Fatty acids	Fatty Acids and Conjugates	Oxo fatty acids	CNP0267906.0
4143	3,5-Diiodotyrosine	1.56	Amino acids and Peptides	Small Peptides	Aminoacids	CNP0246057.1
4586	Conidendrin	1.59	Shikimates and Phenylpropanoids	Lignans	Arylnaphthalene and aryltetralin lignans	CNP0205367.1
5036	Caffeine	1.60	Alkaloids	Pseudoalkaloids	Purine alkaloids	CNP0228556.0
5113	Pomiferin	1.33	Shikimates and	Isoflavonoids	Isoflavanones	CNP0201090.0

			Phenylpropanoids			
5159	Chartreusin	1.23	Shikimates and Phenylpropanoids	Coumarins	Simple coumarins	CNP0162432.1
5366	Noscapine	1.08	Alkaloids	Tyrosine alkaloids	Isoquinoline alkaloids	CNP0236036.1
5379	Stearyl Alcohol	1.64	Fatty acids	Fatty acyls	Fatty alcohols	CNP0192475.0
5863	4(1H)-Quinazolinone	1.55	Alkaloids	Anthranilic acid alkaloids	Quinazoline alkaloids	CNP0490983.0
5897	Vulpinic Acid	1.34	Shikimates and Phenylpropanoids	Diazotetronic acids and derivatives	Pulvinones	CNP0179149.0
6435	(-)-Sandaracopimaric acid	1.45	Terpenoids	Diterpenoids	Pimarane and Isopimarane diterpenoids	CNP0178418.6
6832	Verbenone	1.47	Terpenoids	Monoterpenoids	Pinane monoterpenoids	CNP0109275.1
7521	Proscillaridin	1.50	Terpenoids	Steroids	Bufadienolides	CNP0222708.1
7524	Veratridine	1.18	Alkaloids	Pseudoalkaloids	Steroidal alkaloids	CNP0212321.2
7525	Scillaren A	1.49	Terpenoids	Steroids	Bufadienolides	CNP0292626.5
7532	Lanatoside A	1.54	Terpenoids	Steroids	Cardenolides	CNP0150344.1
7533	Lanatoside C	1.57	Terpenoids	Steroids	Cardenolides	CNP0280265.1
7535	Lanatoside B	1.57	Terpenoids	Steroids	Cardenolides	CNP0318603.2
7606	Allantoin	1.53	Alkaloids	Pseudoalkaloids	Purine alkaloids	CNP0273828.1
7616	cis-Aconitic Acid	1.60	Fatty acids	Fatty Acids and Conjugates	Dicarboxylic acids	CNP0184949.0
7652	Adenosine	1.60	Carbohydrates	Nucleosides	Purine nucleos(t)ides	CNP0216822.2
7668	Aleuritic Acid	1.14	Fatty acids	Fatty Acids and Conjugates	Hydroxy fatty acids	CNP0159651.1
8519	Khellin	1.26	Polyketides	Chromanes	Chromanes	CNP0388833.0
8625	Lawsone	1.59	Polyketides	Naphthalenes	Naphthoquinones	CNP0303808.0
8661	Brasilin	1.40	Shikimates and	Isoflavonoids	-	CNP0384719.1

			Phenylpropanoids			
8751	Crotonic Acid	1.50	Fatty acids	Fatty Acids and Conjugates	Unsaturated fatty acids	CNP0379669.0
8797	Deoxycholic Acid	1.52	Terpenoids	Steroids	Cholane steroids	CNP0207602.1
8973	Fencholic acid	1.42	Terpenoids	Monoterpenoids	Menthane monoterpenoids	CNP0128029.1
9170	N-Deacetylthiocolchicine	1.43	Alkaloids	Tyrosine alkaloids	Phenethylisoquinoline alkaloids	CNP0205939.1
9248	alpha-D-Galacturonic acid	1.49	Carbohydrates	Saccharides	Monosaccharides	CNP0171089.2
9665	Fumagillol	1.24	Terpenoids	Sesquiterpenoids	-	CNP0225350.2
9699	Estrone	1.46	Terpenoids	Steroids	Estrane steroids	CNP0395406.1
10105	(+)-Xylopinine	1.51	Alkaloids	Tyrosine alkaloids	Isoquinoline alkaloids	CNP0218973.2
11440	Protopine Hydrochloride	1.23	Alkaloids	Tyrosine alkaloids	Isoquinoline alkaloids	CNP0214801.0
11866	4-Methoxychalcone	1.52	Shikimates and Phenylpropanoids	Flavonoids	Chalcones	CNP0214842.0
11905	Lapachol	1.54	Polyketides	Naphthalenes	Naphthoquinones	CNP0076389.0
11926	Aristolochic Acid	1.23	Alkaloids	Tyrosine alkaloids	Aporphine alkaloids	CNP0490967.0
12097	Resorufin	1.63	Alkaloids	Anthranilic acid alkaloids	Phenoxazine alkaloids	CNP0214685.0
12444	2-oxohexahydro-2H-3,5-methanocyclopenta[b]furan-7-carboxylic acid	1.54	Terpenoids	Monoterpenoids	Iridoids monoterpenoids	CNP0267178.0
12865	Quinine (Hydrobromide)	1.49	Alkaloids	Tryptophan alkaloids	-	CNP0191532.21
13123	Creatinine	1.43	Alkaloids	Histidine alkaloids	Imidazole alkaloids	CNP0479644.0
13252	Chlortetracycline hydrochloride	1.66	Polyketides	Polycyclic aromatic polyketides	Tetracyclines	CNP0320283.1
14135	Alloevodionol	1.44	Polyketides	-	-	CNP0256322.0
14664	Xanthine	1.48	Alkaloids	Pseudoalkaloids	Purine alkaloids	CNP0583155.0

14665	Hypoxanthine	1.47	Alkaloids	Pseudoalkaloids	Purine alkaloids	CNP0551907.0
14974	Glaucarubol	1.48	Terpenoids	Triterpenoids	Quassinoids	CNP0216135.2
14975	Glaucarubina	1.54	Terpenoids	Triterpenoids	Quassinoids	CNP0223863.1
15307	Quininone	1.54	Alkaloids	Tryptophan alkaloids	-	CNP0172869.0
15624	Pubescine	1.44	Alkaloids	Pseudoalkaloids	Steroidal alkaloids	CNP0228098.1
15780	Amygdalin	1.70	Amino acids and Peptides	Amino acid glycosides	Cyanogenic glycosides	CNP0277873.1
16631	Protocatechuic Acid	1.51	Shikimates and Phenylpropanoids	Phenolic acids (C6-C1)	Simple phenolic acids	CNP0348948.0
18334	Cinerubine A	1.51	Polyketides	Polycyclic aromatic polyketides	Anthracyclines	CNP0312797.2
18805	Gamma-Thujaplicin	1.48	Terpenoids	Monoterpenoids	Menthane monoterpenoids	CNP0218976.0
19028	Flavone	1.46	Shikimates and Phenylpropanoids	Flavonoids	Flavones	CNP0216699.0
19038	1-(2-Hydroxy-3,4,6-trimethoxyphenyl)-3-(4-methoxyphenyl)-2-propen-1-one	1.45	Shikimates and Phenylpropanoids	Flavonoids	Chalcones	CNP0180867.0
19509	Yohimbine Hydrochloride	1.34	Alkaloids	Tryptophan alkaloids	Yohimbine-like alkaloids	CNP0305326.1
19990	Streptovaricin C	1.43	Polyketides	Macrolides	Ansa macrolides	CNP0165743.1
20103	Gallic Acid	1.42	Shikimates and Phenylpropanoids	Phenolic acids (C6-C1)	Gallotannins	CNP0133244.0
20264	5'-adenylic acid	1.40	Carbohydrates	Nucleosides	Purine nucleos(t)ides	CNP0216781.2
21725	Thermopsine	1.38	Alkaloids	Lysine alkaloids	Pyridine alkaloids	CNP0418808.5
21728	Sempervirine nitrate	1.42	Alkaloids	Tryptophan alkaloids	Carboline alkaloids	CNP0533132.0
22070	Pyrethrosin	1.66	Terpenoids	Sesquiterpenoids	Germacrane sesquiterpenoids	CNP0215514.1

22842	Coumestrol	1.66	Shikimates and Phenylpropanoids	Isoflavonoids	Coumestan	CNP0328371.0
22939	Aspergillic acid	1.17	Alkaloids	Tetramate alkaloids	Pyrazine and Piperazine alkaloids	CNP0168796.2
23615	Deoxyuridine	1.24	Carbohydrates	Nucleosides	Pyrimidine nucleos(t)ides	CNP0363563.1
23878	(+-)-Dihydromethysticin	1.24	Shikimates and Phenylpropanoids	Styrylpyrones	Kavalactones and derivatives	CNP0211210.1
23969	Himbafcine	1.57	Alkaloids	Lysine alkaloids	Piperidine alkaloids	CNP0226828.1
24819	beta-Peltatin	1.26	Shikimates and Phenylpropanoids	Lignans	Arylnaphthalene and aryltetralin lignans	CNP0249641.1
24872	Lomatiol	1.27	Polyketides	Naphtalenes	Naphthoquinones	CNP0203832.0
24951	Strychnine N-oxide	1.38	Alkaloids	Tryptophan alkaloids	Aspidosperma-Iboga hybrid type (Vinca alkaloids)	CNP0571331.2
26254	L-Quebrachitol	1.12	Carbohydrates	Polyols	Cyclitols	CNP0201870.17
26258	Rotenone	1.68	Shikimates and Phenylpropanoids	Isoflavonoids	Retenoids	CNP0370102.2
26271	Cyclophosphamide	1.66	Alkaloids	-	-	CNP0248991.0
26326	Beta-Lapachone	1.67	Polyketides	Naphthalenes	Naphthoquinones	CNP0206923.0
26327	alpha-Lapachone	1.16	Polyketides	Naphthalenes	Naphthoquinones	CNP0215210.0
27425	L-citrulline	1.16	Amino acids and Peptides	Small Peptides	Aminoacids	CNP0209633.1
28841	Oxonate	1.19	Alkaloids	Pseudoalkaloids	Pteridine alkaloids	CNP0591355.0
29854	Isorescinnamine, dihydro-	1.61	Alkaloids	Tryptophan alkaloids	Yohimbine-like alkaloids	CNP0136376.0
30238	Dihydroxymaleic acid	1.20	Fatty acids	Fatty Acids and Conjugates	Dicarboxylic acids	CNP0237095.0

30552	alpha-Mangostin	1.62	Shikimates and Phenylpropanoids	Xanthones	Plant Xanthones	CNP0158137.0
30625	Lasiocarpine	1.31	Alkaloids	Ornithine alkaloids	Pyrrolizidine alkaloids	CNP0278473.3
31048	Neohesperidin	1.69	Shikimates and Phenylpropanoids	Flavonoids	Flavanones	CNP0320778.1
31754	Anthragallol	1.11	Polyketides	Polycyclic aromatic polyketides	Anthraquinones and anthrones	CNP0218669.0
31867	Norlobaridone	1.74	Polyketides	Aromatic polyketides	Depsidones	CNP0214538.0
32192	Bicuculline	1.76	Alkaloids	Tyrosine alkaloids	Isoquinoline alkaloids	CNP0191492.1
32743	Acetoxycycloheximide	1.20	Alkaloids	Lysine alkaloids	Piperidine alkaloids	CNP0138673.3
32944	Cephaeline hydrochloride	1.29	Alkaloids	Tyrosine alkaloids	Isoquinoline alkaloids	CNP0105433.2
32979	Isocorydine	1.68	Alkaloids	Tyrosine alkaloids	Aporphine alkaloids	CNP0211797.1
32982	Curcumin	1.68	Shikimates and Phenylpropanoids	Diarylheptanoids	Linear diarylheptanoids	CNP0337918.0
32984	Cryptopine	1.24	Alkaloids	Tyrosine alkaloids	Protopine alkaloids	CNP0370354.0
33410	Colchicine, N-deacetyl-N-benzoyl-	1.25	Alkaloids	Tyrosine alkaloids	Phenethylisoquinoline alkaloids	CNP0261172.1
34202	Cholan-24-oic acid, 3-(acetyloxy)-4,4,14-trimethyl-7,11-dioxo-, methyl ester, (3beta,5alpha)-	1.34	Terpenoids	Triterpenoids	Cycloartane triterpenoids	CNP0194951.1
34552	(5-Oxidooctahydro-2h-quinolizin-4-yl)methanol	1.11	Alkaloids	Lysine alkaloids	Quinolizidine alkaloids	CNP0242688.0
34758	Melicopine	1.12	Alkaloids	Anthranilic acid alkaloids	Acridone alkaloids	CNP0420995.0

35550	3,4-Dihydropapaverine oxalate	1.07	Alkaloids	Tyrosine alkaloids	Isoquinoline alkaloids	CNP0302558.0
35611	Solanine, hydrochloride	1.69	Alkaloids	Pseudoalkaloids	Steroidal alkaloids	CNP0213318.0
**35676**	**Purpurogallin**	0.78	**Polyketides**	**-**	**-**	**CNP0550010.0**
36294	Evodinnol	1.16	Polyketides	Meroterpenoids	Merohemiterpenoids	CNP0277780.0
36351	(+-)-Tetrahydroberberine	1.60	Alkaloids	Tyrosine alkaloids	Isoquinoline alkaloids	CNP0410168.2
36354	Desacetylcolchicine d-tartrate	1.29	Alkaloids	Tyrosine alkaloids	Isoquinoline alkaloids	CNP0410168.2
36398	Quercetin, dihydro-	1.68	Alkaloids	Tyrosine alkaloids	Phenethylisoquinoline alkaloids	CNP0184648.1
36407	Picropodophyllin	1.16	Shikimates and Phenylpropanoids	Lignans	Arylnaphthalene and aryltetralin lignans	CNP0213414.5
36437	[(1R,2S,4E,8Z,12R,13R)-12-hydroxy-4,8,12-trimethyl-16-methylidene-15-oxo-14-oxabicyclo[11.3.1]heptadeca-4,8-dien-2-yl] acetate	1.23	Terpenoids	Diterpenoids	Cembrane diterpenoids	CNP0118712.3
36508	Limonin	1.20	Terpenoids	Triterpenoids	Limonoids	CNP0188078.1
36693	Marrubiin	1.20	Terpenoids	Diterpenoids	Labdane diterpenoids	CNP0107084.1
38010	Ethyl Chaulmoograte	1.17	Fatty acids	Fatty esters	Wax monoesters	CNP0391273.1
38270	Antibiotic A-649	1.38	Polyketides	-	-	CNP0239587.0
42038	(3S,3aS,5aS,8E,9bS)-8-hydroxyimino-3,5a,9-trimethyl-3a,4,5,9b-tetrahydro-3H-benzo[g][1]be	1.59	Terpenoids	Sesquiterpenoids	Eudesmane sesquiterpenoids	CNP0162290.1

	nzofuran-2-one					
43338	Gamma-Thujaplicin	1.10	Terpenoids	Monoterpenoids	Menthane monoterpenoids	CNP0218976.0
43339	Nootkatin	1.13	Terpenoids	Monoterpenoids	~	CNP0572760.0
43871	Tropine	1.11	Alkaloids	Ornithine alkaloids	Tropane alkaloids	CNP0346043.1
44138	1-O-phosphonohexopyranose	1.17	Carbohydrates	Saccharides	Monosaccharides	CNP0189411.10
44175	2,5-Dihydroxy-3,6-diphenyl-2,5-cyclohexadiene-1,4-dione	1.16	Shikimates and Phenylpropanoids	Terphenyls	p-Terphenyls	CNP0292824.0
45383	Streptonigrin	1.73	Alkaloids	Nicotinic acid alkaloids	Pyridine alkaloids	CNP0176065.0
45384	Methyl Streptonigrin	1.14	Alkaloids	Nicotinic acid alkaloids	Pyridine alkaloids	CNP0293149.0
45923	Methoxsalen	1.62	Shikimates and Phenylpropanoids	Coumarins	Furocoumarins	CNP0214931.0
46709	L-leucine	1.13	Amino acids and Peptides	Small Peptides	Aminoacids	CNP0201141.1
46728	Crassin, acetate	1.22	Terpenoids	Diterpenoids	Cembrane diterpenoids	CNP0118712.1
47147	2,2'-Bipyrrole, 4-methoxy-5-((5-methyl-4-pentyl-2H-pyrrol-2-ylidene)methyl)-	1.17	Alkaloids	Proline alkaloids	Prodigiosins	CNP0141256.0
50131	3,4-Dihydroxy-3-(hydroxymethyl)-6,7-dimethoxy-1-(3,4,5-trimethoxyphenyl)-1,2,3,4-tetrahydronaphthalene-2-carboxylic acid	1.23	Shikimates and Phenylpropanoids	Lignans	Arylnaphthalene and aryltetralin lignans	CNP0217094.0
50132	3a,9a-Dihydroxy-	1.22	Shikimates and	Lignans	Arylnaphthalene and	CNP0354336.0

	6,7-dimethoxy-9-(3,4,5-trimethoxyphenyl)-3a,4,9,9a-tetrahydronaphtho[2,3-c]furan-1(3h)-one		Phenylpropanoids		aryltetralin lignans	
50393	Flavanone	1.32	Shikimates and Phenylpropanoids	Flavonoids	Flavanones	CNP0234960.2
51001	Carbomycin	1.62	Polyketides	Macrolides	Tylosins	CNP0369116.7
51351	Flavokawain B	1.09	Shikimates and Phenylpropanoids	Flavonoids	Chalcones	CNP0169290.0
56410	Porfiromycin	1.10	Alkaloids	Mitomycin derivatives	Mitomycins	CNP0342201.1
56464	Aconitine	1.65	Alkaloids	Pseudoalkaloids	Terpenoid alkaloids	CNP0240724.5
58368	Fumagillin dicyclohexylamine salt	1.67	Polyketides	-	-	CNP0212264.0
59258	Quinate	1.16	Shikimates and Phenylpropanoids	Organic oxygen compounds	Organooxygen compounds	CNP0205647.2
**59263**	**Digallic Acid**	0.86	**Polyketides**	**Phenylpropanoids and polyketides**	**Depsides and depsidones**	**CNP0137334.0**
59729	Sparsomysin	1.15	Alkaloids	-	-	CNP0544877.1
60387	Tylocrebrine	1.71	Alkaloids	Benzenoids	Phenanthrenes and derivatives	CNP0272533.1
61809	Pamine	1.30	Alkaloids	Ornithine alkaloids	Tropane alkaloids	CNP0490942.1
62709	THOS Streptonigrin	1.68	Alkaloids	Organoheterocyclic compounds	Pyridines and derivatives	CNP0324448.0
63701	Toyocamycin nucleoside	1.54	Carbohydrates	Nucleosides, nucleotides, and analogues	yrrolopyrimidine nucleosides and nucleotides	CNP0238626.1
67574	Vincristine Sulfate	1.54	Alkaloids	Tryptohan alkaloids	Aspidosperma type	CNP0250330.0
71795	Ellipticine	1.62	Alkaloids	Organoheterocyclic compounds	Indoles and derivatives	CNP0419453.0

72116	Pseudoyohimbine	1.58	Alkaloids	Alkaloids and derivatives	orynanthean-type alkaloids	CNP0305326.7
76022	Thaspine, acetate	1.55	Shikimates and Phenylpropanoids	Phenylpropanoids and polyketides	Coumarins and derivatives	CNP0214130.0
82151	Daunorubicin	1.60	Polyketides	Phenylpropanoids and polyketides	Anthracyclines	CNP0221000.3
85236	Helenalin	1.58	Terpenoids	Lipids and lipid-like molecules	Prenol lipids	CNP0201485.3
85239	Parthenicin	1.59	Terpenoids	Lipids and lipid-like molecules	Prenol lipids	CNP0148754.1
87511	Stictic Acid	1.28	Polyketides	Aromatic polyketides	Depsidones	CNP0231377.1
89671	Brefeldin A	1.59	Polyketides	Organic acids and derivatives	Carboxylic acids and derivatives	CNP0170362.1
94600	Camptothecin	1.60	Alkaloids	Alkaloids and derivatives	Camptothecins	CNP0187308.1
96911	Lumichrome	1.65	Alkaloids	Organoheterocyclic compounds	Pteridines and derivatives	CNP0210205.0
105388	Fungichromin	1.59	Polyketides	Macrolides	Polyene macrolides	CNP0244118.0
114344	Ascochitine	1.55	Polyketides	Benzenoids	Benzene and substituted derivatives	CNP0134387.1
118343	Radicinin	1.51	Polyketides	Organic oxygen compounds	Organooxygen compounds	CNP0219600.1
122023	Valinomycin	1.53	Amino acids and Peptides	Organic acids and derivatives	Peptidomimetics	CNP0205805.3
122224	Sporidesmolide I	1.58	Amino acids and Peptides	Oligopeptides	Cyclic peptides	CNP0570253.0
122750	(13-Hydroxy-8,14,19-trimethoxy-4,10,12,16-tetramethyl-3,20,22-trioxo-2-azabicyclo[16.3.1]docosa-1(21),4,6,10,18-pentaen-9-yl) carbamate	1.54	Polyketides	Macrolides	Ansa macrolides	CNP0233043.0

122819	Teniposide	1.59	Shikimates and Phenylpropanoids	Lignans, neolignans and related compounds	Lignan lactones	CNP0311818.1
129536	Picrotin	1.62	Terpenoids	Organoheterocyclic compounds	Furopyrans	CNP0211392.1
133100	Rifamycin Sodium	1.59	Polyketides	Organoheterocyclic compounds	Naphthofurans	CNP0173777.2
145118	Lankacidin C	1.58	Terpenoids	Diterpenoids	Cembrane diterpenoids	CNP0213988.0
150817	Stamycin (Nystatin)	1.60	Polyketides	Organic acids and derivatives	Hydroxy acids and derivatives	CNP0272517.3
153858	Maytansine	1.62	Polyketides	Organic acids and derivatives	Carboxylic acids and derivatives	CNP0481478.1
157035	Germacra-1(10),11(13)-dien-12-oic acid, 4,5-alpha-epoxy-6-beta-hydroxy-, gamma-lactone (Parthenolide)	1.60	Terpenoids	Lipids and lipid-like molecules	Prenol lipids	CNP0212480.6
169627	Streptoval C	1.57	Polyketides	Benzenoids	Naphthalenes	CNP0269555.0
176503	Fastigillin B	1.51	Terpenoids	Sesquiterpenoids	Pseudoguaiane sesquiterpenoids	CNP0138678.0
177406	Antibiotic X-536A	1.52	Polyketides	Polyethers	Open-chain polyketides	CNP0326860.0
177858	Virginiamycin S1	1.57	Amino acids and Peptides	Organic acids and derivatives	Peptidomimetics	CNP0207716.2
180515	(7E)-2-acetyl-2,3,4,5-tetrahydrooxonine-6,9-dione	1.58	Polyketides	Cyclic polyketides	-	CNP0128252.0
209870	Antibiotic A-31438	1.48	Polyketides	Macrolides	Erythromycins	CNP0213862.0
210236	Crassin (Cetylpyridinium chloride)	1.59	Alkaloids	Organoheterocyclic compounds	Pyridines and derivatives	CNP0550857.0
226080	Sirolimus	1.54	Polyketides	Macrolides	Ascomycins and Rapamycins	CNP0175721.3
250429	CID 317610	1.59	Terpenoid	Diterpenoids	labdane diterpenoids	CNP0393616.0

250430	Gangetin	1.64	Shikimates and Phenylpropanoids	Isoflavonoids	Pterocarpan	CNP0225385.1
255109	Ipi-493	1.59	Polyketides	Macrolides	Ansa macrolides	CNP0222745.1
olecule is	(2R,3S)-3-(6-amino-9H-purin-9-yl)nonan-2-ol	1.61	Alkaloids	Pseudoalkaloids	Purine alkaloids	CNP0175714.2
270914	Photobarbatusin I	1.56	Terpenoids	Diterpenoids	Cycloabietane diterpenoids	CNP0154312.1
284200	Batyl Alcohol	1.63	Fatty acids	Glycerolipids	Monoacylgycerol	CNP0135319.1
284437	Quadrone	1.58	Terpenoids	Sesquiterpenoids	Quadrane sesquiterpenoids	CNP0215604.2
285116	Siomycin A	1.64	Aminoacids and peptides	Oligopeptides	RiPPs-Thiopeptides	CNP0237719.0
287088	Physalin B	1.60	Terpenoids	Steroids	Ergostane steroids	CNP0168775.5
292567	Nigericin sodium salt (from Streptomyces hygroscopicus)	1.55	Plyketides	Polyethers	Polyether ionophores	CNP0217597.1
301683	Daphnetin diacetate	1.62	Shikimates and Phenylpropanoids	Coumarins	Simple coumarins	CNP0224297.0
302289	(+)-Alantolactone	1.69	Terpenoids	Sesquiterpenoids	Eudesmane sesquiterpenoids	CNP0288350.0
305222	Cytochalasin-H	1.67	Alkaloids	Pseudoalkaloids	Cytochalasan alkaloids	CNP0303748.3
307981	Lonchocarpic acid	1.65	Shikimates and Phenylpropanoids	Coumarins	Pyranocoumarins	CNP0223446.0
325014	Bactobolin	1.75	Shikimates and Phenylpropanoids	Coumarins	Isocoumarins	CNP0216238.1
325319	Didemnin A	1.67	Aminoacids and Peptides	Oligopeptides	Cyclic peptides	CNP0529731.1
330753	Baccatin III	1.64	Terpenoids	Diterpenoids	taxane diterpenoids	CNP0358369.2

332598	Rhizoxin	1.69	Aminoacids and Peptides	Macrolides	Rhizoxins	CNP0215436.4
332876	Hispanolone	1.62	Terpenoids	Diterpenoids	Labdane diterpenoids	CNP0213253.1
333856	Tetrocarcin A, sodium salt	1.69	polyketides	Macrolides	Spirotetronate macrolides	CNP0569486.3
337783	methyl (1aS,1bS,3aR,4R,7aS,9aS)-1b-hydroxy-4,7a-dimethyl-9-oxo-9a-propan-2-yl-2,3,3a,5,6,7-hexahydro-1aH-phenanthro[1,2-b]oxirene-4-carboxylate	1.64	Terpenoids	Diterpenoids	Abeoabietane diterpenoids	CNP0198056.1
339555	Bryostatin I	1.62	Polyketides	Macrolides	Bryostatins	CNP0215064.1
345647	Chaetochromin A	1.45	Polyketides	Naphtalenes	Bisnaphtalenes	CNP0197429.1
349438	4-Ipomeanol	1.67	Terpenoids	-	-	CNP0135487.0
350085	Medicarpin	1.69	Shikimates and Phenylpropanoids	Isoflavonoids	Pterocarpan	CNP0214655.2
361902	Sequiterpene lactone 326	1.68	Terpenoids	Sesquiterpenoids	Pseudoguaiane sesquiterpenoids	CNP0351870.0
369397	Artemisinin	1.61	Terpenoids	Sesquiterpenoids	Artemisin	CNP0216348.9
375294	Confertifoline	1.55	Terpenoids	Sesquiterpenoids	Drimane sesquiterpenoids	CNP0240386.0
376248	5-Methoxy-2,12-diphenyl-3,4-dihydro-2H,9H-dipyrano(2,3,4-kl:2',3'-a)xanthen-9-one	1.73	Shikimates and Phenylpropanoids	Flavonoids	Flavans	CNP0197677.1
382796	benzyl 4'-(1,3-dioxoisoindol-2-yl)-2,2-dimethyl-1,5'-dioxospiro[3aH-	1.55	Alkaloids	Anthranilic acid alkaloids	Quinazoline alkaloids	CNP0218410.0

	imidazo[1,2-a]indole-4,2'-oxolane]-3-carboxylate					
400978	Illudin M	1.62	Terpenoids	Sesquiterpenoids	Illudane sesquiterpenoids	CNP0184977.1
401005	Pleurotine	1.60	Terpenoids	-	-	CNP0218715.0
407286	Ellagic Acid	1.44	Shikimates and Phenylpropanoids	Phenolic acids (C6-C1)	Gallotannins	CNP0224900.0
407306	Corynanthine	1.62	Alkaloids	Tryptophan alkaloids	Yohimbine-like alkaloids	CNP0305326.14
614552	L-1,6-Diepicastanospermine	1.48	Alkaloids	Lysine alkaloids	indolizidine alkaloids	CNP0239914.0
637086	Pentoxifylline	1.57	Alkaloids	Pseudoalkaloids	Purine alkaloids	CNP0155893.0
661755	(-)-Michellamine B	1.46	Alkaloids	Tyrosine alkaloids	Isoquinoline alkaloids	CNP0168638.2
719655	Fumitremorgin C	1.69	Alkaloids	Peptide alkaloids	Indole diketopiperazine alkaloids (L-Trp, L-Pro)	CNP0208645.1

[a] NSC is a unique identifier assigned by the NCI Developmental Therapeutics Program (DTP) to catalog and track chemical compounds. In this study, 204 compounds from the Natural Product Set IV of the NCI DTP were evaluated for their ability to inhibit Zika virus (ZIKV) RNA-dependent RNA polymerase (RdRp). This table lists the NSC numbers of all compounds investigated. More information about the compound plates is available at: https://wiki.nci.nih.gov/display/ncidtpdata/compound+sets.

[b] The complete chemical names were obtained from the NCBI PubChem website: https://pubchem.ncbi.nlm.nih.gov/.

[c] ZIKV RdRp activity as measured by Malachite Green colorimetric assay in presence of all the natural compounds investigated in this study.

[d] The compounds analyzed in this study were classified according to pathway, superclass, and class using data from COCONUT (COlleCtion of Open Natural prodUcTs), a comprehensive resource of elucidated and predicted natural products compiled from open-access databases (available at https://coconut.naturalproducts.net)[1].

[e] Each compound was referenced by its unique COCONUT identifier (COCONUT ID, format: CNPXXXXXXX), which links to the corresponding entry in the COCONUT database.

[f] The two compounds, purpurogallin and digallic acid, that inhibited ZIKV RdRp in the malachite green assay are indicated in bold.

Table S2. List of all natural products examined in our study and identified as antioxidants.

NSC Number	Compound	Evidence
20103	Gallic Acid	Gallic acid and its derivatives are known for their antioxidant properties; they show strong Reactive Oxygen Species (ROS) scavenging (96% activity)[2].
59263	Digallic acid	Digallic acid exhibits potent antioxidant properties, showing strong free radical scavenging activity (≈99% $ABTS^{+}$ inhibition)[3].
32982	Curcumin	Curcumin and its derivatives are widely employed as antioxidants and have studied free radical scavenging activities [4].
16631	Protocatechuic Acid	Described as a perfect peroxyl radical scavenger and good anti-radical protector[5]; enhances antioxidant enzymes[6]
22842	Coumestrol	Coumestrol exhibits DPPH radical scavenging comparable to BHT and is considered a promising antioxidant molecule [7].
36398	Quercetin, dihydro-	Taxifolin (dihydroquercetin) has superior antioxidant capacity and scavenges multiple radicals [8].
30552	Mangostin	α-Mangostin is known for its robust antioxidant activity and beneficial properties as an antioxidant [9].
2150	o-Vanillin	o-Vanillin reduces malondialdehyde and ROS levels, demonstrating potent ROS scavenging and protective properties [10].
407286	Ellagic Acid	Ellagic acid has powerful free radical scavenging activity and is an efficient inhibitor of lipid peroxidation [11].
35676	Purpurogallin	Purpurogallin, a phenolic compound, is known for its high antioxidant properties in plant-based materials [12].
87511	Stictic Acid	Stictic acid is described as having apoptotic, cytotoxic and antioxidant activities [13].

51351	Flavokawain B	Flavokawain B is a natural chalcone shown to possess antioxidant, anti-inflammatory and anticancer properties [14].
36693	Marrubiin	Marrubiin and its extracts display antioxidant and antigenotoxic properties [15].
36508	Limonin	Limonin shows strong antioxidant activity, with radical scavenging abilities surpassing vitamin C; inhibits oxygen free radical generation [16].
301683	Daphnetin diacetate	Daphnetin (coumarin derivative) has diverse bioactivities including antioxidant effects; prevents oxidative stress and exhibits Nrf2-dependent antioxidant activity [17].

The compounds shown in this table have all been shown to possess antioxidant activity. Only two of them—digallic acid and purpurogallin—are the compounds that inhibit the polymerase activity of ZIKV RdRp, as determined by the malachite green assay. Thus, the inhibition of the polymerase activity of ZIKV RdRp is likely not a consequence of their antioxidant activity.

REFERENCES

(1) Chandrasekhar, V.; Rajan, K.; Kanakam, Sri Ram S.; Sharma, N.; Weißenborn, V.; Schaub, J.; Steinbeck, C. COCONUT 2.0: a comprehensive overhaul and curation of the collection of open natural products database. *Nucleic Acids Research* **2025/01/06**, *53* (D1).
(2) Alhyari, D.; Qinna, N. A.; Sheldrake, H. M.; Kantamneni, S.; Ghanem, B. Y.; Paluch, K. J.; Alhyari, D.; Qinna, N. A.; Sheldrake, H. M.; Kantamneni, S.; et al. Antioxidant, Anti-Inflammatory, and Oral Bioavailability of Novel Sulfonamide Derivatives of Gallic Acid. *Antioxidants 2025, Vol. 14, Page 374* **2025-03-21**, *14* (4).
(3) Bhouri, W.; Derbel, S.; Skandrani, I.; Boubaker, J.; Bouhlel, I.; Sghaier, M. B.; Kilani, S.; Mariotte, A. M.; Dijoux-Franca, M. G.; Ghedira, K.; et al. Study of genotoxic, antigenotoxic and antioxidant activities of the digallic acid isolated from Pistacia lentiscus fruits. *Toxicology in Vitro* **2010/03/01**, *24* (2).
(4) Alisi, I. O.; Uzairu, A.; Abechi, S. E.; Idris, S. O. Evaluation of the antioxidant properties of curcumin derivatives by genetic function algorithm. *Journal of Advanced Research* **2018/07/01**, *12*.
(5) Zhang, S.; Gai, Z.; Gui, T.; Chen, J.; Chen, Q.; Li, Y. Antioxidant Effects of Protocatechuic Acid and Protocatechuic Aldehyde: Old Wine in a New Bottle. *Evidence-Based Complementary and Alternative Medicine* **2021/01/01**, *2021* (1).
(6) Varì, R.; D'Archivio, M.; Filesi, C.; Carotenuto, S.; Scazzocchio, B.; Santangelo, C.; Giovannini, C.; Masella, R. Protocatechuic acid induces antioxidant/detoxifying enzyme expression through JNK-mediated Nrf2 activation in murine macrophages. *The Journal of Nutritional Biochemistry* **2011/05/01**, *22* (5).
(7) Koirala, P.; Seong, S. H.; Jung, H. A.; Choi, J. S.; Koirala, P.; Seong, S. H.; Jung, H. A.; Choi, J. S. Comparative Evaluation of the Antioxidant and Anti-Alzheimer's Disease Potential of Coumestrol and Puerarol Isolated from Pueraria lobata Using Molecular Modeling Studies. *Molecules 2018, Vol. 23, Page 785* **2018-03-28**, *23* (4).
(8) Liu, Y.; Shi, X.; Tian, Y.; Zhai, S.; Liu, Y.; Xiong, Z.; Chu, S. Frontiers | An insight into novel therapeutic potentials of taxifolin. *Frontiers in Pharmacology* **2023/05/12**, *14*.
(9) Górecka, H.; Guźniczak, M.; Buzalewicz, I.; Ulatowska-Jarża, A.; Korzekwa, K.; Kaczorowska, A.; Górecka, H.; Guźniczak, M.; Buzalewicz, I.; Ulatowska-Jarża, A.; et al. Alpha-Mangostin: A Review of Current Research on Its Potential as a Novel Antimicrobial and Anti-Biofilm Agent. *International Journal of Molecular Sciences 2025, Vol. 26, Page 5281* **2025-05-30**, *26* (11).
(10) Wang, J.; An, W.; Wang, Z.; Zhao, Y.; Han, B.; Tao, H.; Wang, J.; Wang, X.; Wang, J.; An, W.; et al. Vanillin Has Potent Antibacterial, Antioxidant, and Anti-Inflammatory Activities In Vitro and in Mouse Colitis Induced by Multidrug-Resistant Escherichia coli. *Antioxidants 2024, Vol. 13, Page 1544* **2024-12-17**, *13* (12).
(11) Tošović, J.; Bren, U.; Tošović, J.; Bren, U. Antioxidative Action of Ellagic Acid—A Kinetic DFT Study. *Antioxidants 2020, Vol. 9, Page 587* **2020-07-06**, *9* (7).
(12) Liao, Y.-C.; Kim, T.; Silva, J. L.; Chen, B.-Y.; Liao, Y.-C.; Kim, T.; Silva, J. L.; Chen, B.-Y. A New Approach for Quantifying Purpurogallin in Brewed Beverages Using LC-MS in Combination with Solid Phase Extraction. *Foods 2022, Vol. 11, Page 1429* **2022-05-16**, *11* (10).
(13) Oran, S.; Sahin, S.; Sahinturk, P.; Ozturk, S.; Demir, C.; Oran, S.; Sahin, S.; Sahinturk, P.; Ozturk, S.; Demir, C.; et al. Antioxidant and Antimicrobial Potential, and HPLC Analysis of Stictic and Usnic Acids of Three Usnea Species from Uludag Mountain (Bursa, Turkey). *Iranian Journal of Pharmaceutical Research : IJPR* **2016/09/07**, *15* (2).
(14) SON, J. H.; CHOI, Y. H.; LEE, S. H.; PAIK, W. H.; RYU, J. K.; KIM, Y.-T. Flavokawain B Inhibits Growth of Cholangiocarcinoma Cells by Suppressing the Akt Pathway. *In Vivo* **2023-05-01**, *37* (3).

(15) Rezgui, M.; Majdoub, N.; Mabrouk, B.; Baldisserotto, A.; Bino, A.; Kaab, L. B. B.; Manfredini, S. Antioxidant and antifungal activities of marrubiin, extracts and essential oil from Marrubium vulgare L. against pathogenic dermatophyte strains. *Journal de Mycologie Médicale* **2020/04/01**, *30* (1).
(16) Jun Yu, ‡; Limin Wang; Rosemary L. Walzem; Edward G. Miller; Leonard M. Pike, a.; Bhimanagouda S. Patil*, ‡. Antioxidant Activity of Citrus Limonoids, Flavonoids, and Coumarins. *Journal of Agricultural and Food Chemistry* **March 1, 2005**, *53* (6).
(17) Xia, Y.; Chen, C.; Liu, Y.; Ge, G.; Dou, T.; Wang, P.; Xia, Y.; Chen, C.; Liu, Y.; Ge, G.; et al. Synthesis and Structure-Activity Relationship of Daphnetin Derivatives as Potent Antioxidant Agents. *Molecules 2018, Vol. 23, Page 2476* **2018-09-27**, *23* (10).
